# Fimbriae-mediated outer membrane vesicle production and invasion of *Porphyromonas gingivalis*

**DOI:** 10.1002/mbo3.221

**Published:** 2014-12-18

**Authors:** Chinmay K Mantri, Chin-Ho Chen, Xinhong Dong, Jeffery Shawn Goodwin, Siddharth Pratap, Victor Paromov, Hua Xie

**Affiliations:** 1 School of Dentistry, Meharry Medical CollegeNashville, Tennessee; 2 Department of Surgery, Duke University Medical CenterDurham, North Carolina; 3 Department of Microbiology and Immunology, Meharry Medical CollegeNashville, Tennessee; 4 Department of Biochemistry and Cancer Biology, Meharry Medical CollegeNashville, Tennessee

**Keywords:** Invasion, outer membrane vesicles, *P. gingivalis*

## Abstract

*Porphyromonas gingivalis* is a keystone periopathogen that plays an essential role in the progress of periodontitis. Like other gram-negative bacteria, the ability of *P. gingivalis* to produce outer membrane vesicles is a strategy used to interact with, and survive within its biological niches. Here we compared the protein components associated with vesicles derived from a fimbriated strain (33277) and an afimbriated strain (W83) of *P. gingivalis* using proteomic analyses. Some well-known virulence factors were identified in vesicles from both strains, such as gingipains and hemagglutinin. In contrast, FimC, FimD, and FimE, minor components of long fimbriae were found exclusively in 33277 vesicles, while proteins with a tetratricopeptide repeat (TPR) domain were unique to W83 vesicles. We found that significantly more 33277 than W83 vesicles were internalized into human oral keratinocytes and gingival fibroblasts. Interestingly, FimA, a well-known adhesin responsible for the attachment and invasion of *P. gingivalis* into host cells, was not essential for the invasive capabilities of *P. gingivalis* vesicles. Rather minor components of long fimbriae were required for an efficient invasive activity of vesicles. The most striking finding was that *P. gingivalis* strains lacking or having a reduced FimA expression showed a significant reduction in vesiculation. These results suggest that production and pathogenicity of *P. gingivalis* vesicles may largely depend on expression of the *fim* locus, and that the integration of vesicle production and pathogenicity with fimbrial expression may allow *P. gingivalis* to confer upon itself certain functional advantages.

## Introduction

Vesicles, generally produced by gram-negative bacteria, are found both on the surfaces of cells and in their surrounding environments (MacDonald and Kuehn 2012). Vesicles have also been isolated from biofilms (Schooling and Beveridge 2006; Toyofuku et al. 2012). Recently, the prevalence of vesicles was found to be relatively abundant in coastal and open-ocean seawater at a level similar to the concentrations of bacteria at these sites, indicating that vesicles are produced not only by biofilm-inhabiting microbes living at high cell density but also by planktonic bacteria in the open ocean (Biller et al. 2014). Bacterial vesicles appear to protect the bacteria from membrane active antibiotics by scavenging them or by acting as decoy to prevent phage attack (Scanlan 2014). These observations suggest that vesicles perform some specific functions related to bacterial survival in ecosystems. Although vesicles are released continuously in many gram-negative bacteria, the levels of vesiculation in *Escherichia coli* appear to be increased under stressful conditions, such as elevated temperatures (McBroom and Kuehn 2007). Therefore, vesicle production may be one of the protective mechanisms utilized by bacteria in hostile environments.

*Porphyromonas gingivalis* is a gram-negative bacterium associated with chronic periodontitis. The organism has been observed to both express vesicles on their cell surface and release them to its environments (Grenier and Mayrand 1987; Smalley and Birss 1987). Consistent with vesicles of other gram-negative bacteria, *P. gingivalis* vesicles are between 50 and 250 nm in diameter but are predominantly around 50 nm. A recent study, using proteomics analyses, reported that protein profiles of *P. gingivalis* vesicles are similar with major outer membrane proteins such as FimA, HagA, and gingipains (Kgp, RgpA, and RgpB) (Imai et al. 2005; Nakao et al. 2014). This suggests that these vesicles may serve as a vehicle for some important virulence factors and proteolytic enzymes of *P. gingivalis*. Since they carry the major outer membrane proteins of *P. gingivalis*, the vesicles have shown the following characteristic features, such as induction of immune responses, coaggregation with other oral bacteria, and host cell invasion. For example, Nakao et al. (2014) reported that these vesicles were highly antigenic, as that absorption of patient sera with vesicles greatly reduced reactivity with whole cells of *P. gingivalis*. An invasive activity of *P. gingivalis* vesicles was also observed. It was reported that after exposure to HeLa and immortalized human gingival epithelial (IHGE) cells, intracellular *P. gingivalis* vesicles were detected within 15 min (Furuta et al. 2009b). This process appears to be a FimA and gingipain dependent, since vesicles of the *fimA* mutant and the *rgp*-null mutant showed a much lower interaction with host cells (Furuta et al. 2009a,b). One of the most recognized features of *P. gingivalis* is its role as a keystone pathogen involved in modulating the virulence of the entire microbial community through interactive communication with other oral pathogens (Hajishengallis and Lamont 2012). Previous studies demonstrated that *P. gingivalis* vesicles are able to enhance the attachment and invasion of *Tannerella forsythia* to epithelial cells, and mediate coaggregation between *Treponema denticola* and *Lachnoanaerobaculum saburreum* and between mycelium-type *Candida albicans* and *Staphylococcus aureus* (Kamaguchi et al. 2003; Inagaki et al. 2006; Grenier 2013). Therefore, an in-depth understanding of vesicles with these distinct features is an important area of not only periodontitis research but also periodontitis-associated systemic diseases.

In order to characterize *P. gingivalis* vesicles, we examined vesicle production and invasive activity in wild-type strains of *P. gingivalis* W83, 33277, and their mutants. The results revealed a fimbrial-mediated vesiculation in *P. gingivalis*. Using confocal microscopy and Fluorescence-activated cell sorting (FACS), we measured and compared the invasive activities of these vesicles toward human oral keratinocytes (HOKs) and gingival fibroblasts (HGFs). We show in this study that minor components (FimC, D, and E) of long fimbriae, but not FimA, and the adhesive domains of gingipains were involved in the invasive activity of the vesicles. The vesicles appeared to translocate across a monolayer of HOKs in a transwell system and invade HGFs in the lower compartment. These findings provide opportunities to identify pathways and mechanisms of vesicles formation and regulation in *P. gingivalis*.

## Experimental Procedures

### Bacterial strains and vesicle preparation

*Porphyromonas gingivalis* strains were grown from frozen stocks in TSB (trypticase soy broth) or on TSB blood agar plates supplemented with yeast extract (1 mg/mL), hemin (5 *μ*g/mL), and menadione (1 *μ*g/mL), and incubated at 37°C in an anaerobic chamber (85% N_2_, 10% H_2_, 5% CO_2_). *Porphyromonas gingivalis* vesicles were prepared as described previously (Furuta et al. 2009b). Briefly, *P. gingivalis* cells were grown to the late exponential phase and growth media were collected by centrifugation at 10,000*g* for 15 min at 4°C and filtered through a 0.22-*μ*m pore size filters (Cell Treat, Shirley, MA) to remove residual bacteria. Vesicles were collected by ultracentrifugation at 126,000*g* for 2 h at 4°C and resuspended in phosphate-buffered saline (PBS) containing 10% glycerol. Bacterial proteins were extracted from the vesicles using a BugBuster® Protein Extraction Reagent (EMD Biosciences, Madison, WI) and protein concentrations were determined with a Protein Assay Kit (Bio-Rad, Hercules, CA).

### Proteomics analysis of *P. gingivalis* vesicles

Vesicles (8.3 *μ*g) were separated by a short-stack (∼1 cm) SDS-PAGE gel and stained with Coomassie Brilliant Blue. Protein bands were excised from the gels and in-gel digested with trypsin (Promega, Madison, WI). Liquid chromatography–tandem mass spectrometry (LC-MS/MS) was performed with the tryptic peptide samples separated over a 90-min gradient run on a C18 nanocolumn (Phenomenex, Torrance, CA) coupled in-line with a LTQ linear ion trap mass spectrometer (Thermo Fisher Scientific, Waltham, MA, USA). The tandem MS/MS spectra were analyzed using Myrimatch Proteomics software against UniProt protein databases for both *P. gingivalis* 33277 and W83 (Tabb et al. 2007). Peptide mass tolerance was set at ±2 Da, and fragment mass tolerance was set at ±1 Da. The parameters for database search were as follows: semitryptic digestion; up to two missed cleavage sites; cysteine carbamidomethylation (+57 Da) as a fixed modification and methionine oxidation (+16 Da) as a variable modification. The search results were filtered to include only proteins with two or more unique peptide spectra and a protein false discovery rate (FDR) of <5%. The relative differences in protein abundance were estimated by spectral counting approach and statistically analyzed using Fisher's exact test with a statistical significance threshold set at a Benjamini–Hochberg corrected *P* < 0.05.

### Western blot analysis

Surface extracts were extracted by sonication of *P. gingivalis* strains grown in TSB for 16 h at 4°C. Surface extracts and vesicles of *P. gingivalis* (0.5 *μ*g) were separated by 12% sodium dodecyl sulfate–polyacrylamide gel electrophoresis (SDS-PAGE) and transferred to nitrocellulose membranes (Invitrogen, Carlsbad, CA) with a Mini Transblot Electrophoretic transfer cell (Bio-Rad) at 100 V for 1 h. The membrane was treated with blocking solution (3% BSA in PBS) for 1 h and incubated with polyclonal anti-FimA or anti-HGP44 (a C-terminal adhesin domain of gingipains) antibodies diluted 1:1,000 for 1 h. After washing with PBS, the membrane was incubated with anti-rabbit horseradish peroxidase–conjugated secondary antibodies for 1 h. The proteins were visualized using an Enhanced Chemiluminescence (GE Healthcare Bio-Sciences Corp, Pittsburgh, PA) and measured using a semiquantitative western blot technique with ImageJ software (NIH, Bethesda, MD).

### Treatment of host cells with *P. gingivalis* vesicles

HGF and HOK cells were purchased from ScienCell Research Laboratories and cultured in specific media, according to the manufacturer's instruction. To infect HGFs with *P. gingivalis* vesicles, HGFs (1 × 10^4^ or 1.6 × 10^5^) were grown overnight in poly-l-lysine coated 35 mm glass bottom dishes (MatTek Corporation, Ashland, MA) or in six-well plates (Corning Incorporated, Corning, NY), and *P. gingivalis* vesicles (500 ng) were then added to the HGF cultures and incubated for 30 min. After removal of the unbound vesicles, HGFs were cultured for another 0, 0.5, 3.5, and 23.5 h, respectively, with fresh media at 37°C, 5% CO_2_. The cytotoxicity of vesicle treatment was determined with a Pierce LDH Cytotoxicity Assay Kit (Thermo Scientific, Waltham, MA, USA).

### Flow cytometry analysis

After being infected with *P. gingivalis* vesicles, HGFs or HOKs were dissociated with trypsin, fixed with 3.8% formaldehyde solution, and permeabilized with a solution containing 0.1% Triton-X 100 and 0.2% BSA in PBS. The cells were then labeled with pan-specific antibodies of *P. gingivalis* and Alexa Fluor 546 donkey anti-rabbit IgG (Invitrogen). Cells were then examined on a BD FACSCalibur flow cytometer, and the data were analyzed with FlowJo software.

### Confocal microscopy

HGFs were grown overnight in poly-l-lysine coated 35 mm glass bottom dishes, and infected with *P. gingivalis* vesicle. After fixing with 3.8% formaldehyde in a sodium phosphate buffer at room temperature for 10 min, HGFs were permeabilized with 0.1% Triton X-100 for 10 min, and blocked with 5% bovine serum albumin in PBS for 1 h. HGFs were then immunostained with pan-specific antibodies of *P. gingivalis* and followed by goat anti-rabbit IgG conjugated to Alex Fluor 546 (Invitrogen). Confocal images were acquired using a Nikon A1R confocal microscope, NIKON CORPORATION, Tokyo, Japan.

### Transmission electron microscopy of *P. gingivalis* vesicles

Transmission electron microscopy of *P. gingivalis* vesicles was conducted as described previously (Chaudhuri S, et al. 2014). *Porphyromonas gingivalis* vesicles (0.5 ng) were suspended in 25 *μ*L ddH_2_O and applied to a Formvar-coated copper grid (200 mesh, Electron microscopy Sciences, Hatfield, PA) for 1 min and were air-dried. The bacterial cells were then negatively stained with 0.5% ammonium molybdate for 60 sec, and observed under a FEI Tecnai T12 transmission electron microscope (Philips, Hillsboro, OR, USA) operated at 100 kV.

### Penetration of vesicles through HOK layers

HOKs (1 × 10^6^) were seeded onto 3-*μ*m pore size polyethylene terephthalate (PET) transwell inserts (Falcon; BD Biosciences, San Jose, CA) and were allowed to settle for 3 h at 37°C under a 5% CO_2_ atmosphere. The transwell inserts were then placed in a six-well plate (Cell Treat) with fresh Oral Keratinocyte Medium and incubated for 8 days in order to obtain a polarized cell layer. When confluent, cell monolayer integrity was measured using a Lucifer Yellow CH kit (Invitrogen). The transwells were removed from their respective wells and placed into wells of a six-well plate covered with HGFs with HGF growth medium.

*Porphyromonas gingivalis* vesicles that translocated through the monolayered HOKs were collected by centrifugation and resuspended in 50 *μ*L ddH_2_O. Vesicles were quantitated with modifications using an enzyme-linked immunosorbent assay (ELISA) as described previously (Chaudhuri et al. 2014). Briefly, Nunc-immuno modules (Nalgene, San Diego, CA, USA) were coated with vesicle proteins (50 *μ*L) for 16 h at 4°C. The modules were blocked with 3% BSA in PBS–Tween-20. A series of dilutions of pan-33277 polyclonal antibodies were then applied to the modules, and incubated for 3 h. After a wash, the modules were incubated with horseradish peroxidase–conjugated antibodies against rabbit IgG (1:3,000; Amersham Biosciences, Waltham, MA, USA) and peroxide substrate (Sigma) was added to each module. Results were read at 450 nm on a Benchmark Plus microplate reader (Bio-Rad). All samples were assayed in triplicate for each sample.

### RNA isolation and qPCR

*Porphyromonas gingivalis* strains were grown to reach an OD_595_ = 1.0 and then harvested by centrifugation and homogenized in Trizol Reagent (Invitrogen). The RNA in the supernatant was then purified using an RNeasy mini spin column (Qiagen, Valencia, CA). Real-time reverse transcript polymerase chain reaction (RT-PCR) analysis was performed by using a QuantiTect SYBR Green RT-PCR Kit (Qiagen) on the iCycler MyiQ™ Real-Time PCR detection system (Bio-Rad) according to the manufacturer's instructions. Primers are listed in Table S1. The expression levels of the investigated genes for the test samples were determined relative to the untreated calibrator sample by using the comparative cycle threshold (*ΔC*_*T*_) method. The *ΔC*_*T*_ were calculated by subtracting the average *C*_*T*_ value of the test sample from the average *C*_*T*_ value of the calibrator sample, and were then used to calculate the ratio between the two by assuming 100% amplification efficiency (Xie and Zheng 2012).

### Statistical analyses

A Student's *t*-test was used to determine statistical significance of the differences in gene expression profiles and production and invasion of vesicles. A *P *<* *0.05 was considered significant. Values are shown ± SD.

## Results

### FimA is required for vesiculation in *P. gingivalis*

**Figure 1:**
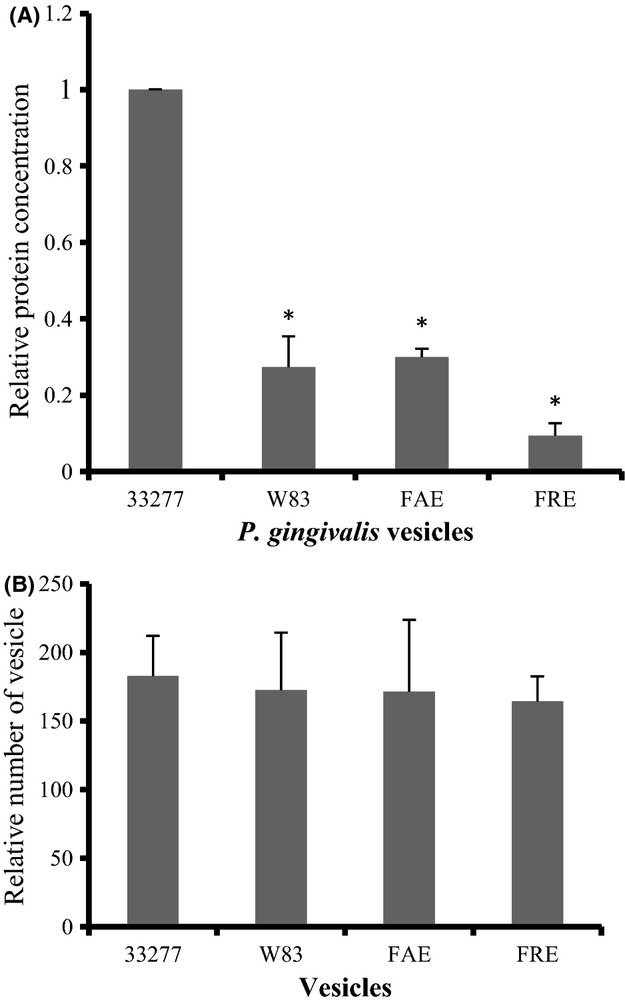
Comparison of vesicle production from *Porphyromonas gingivalis* strains. Vesicles were isolated from the 30 mL of growth media for *P. gingivalis* 33277, *fimA* mutant (FAE), *fimR* mutant (FRE), and an afimbriated strain (W83). (A) Protein concentrations of vesicle suspensions were determined using a Bio-Rad Protein Assay Kit, and each bar presents the relative protein concentrations of vesicles isolated from each *P. gingivalis* strain compared to that of *P. gingivalis* 33277 (as 1 unit). Error bars represent standard deviations (*n *=* *3). An asterisk indicates a significant difference between the protein concentration of vesicles of a given *P. gingivalis* strain compared to *P. gingivalis* 33277 (*P <* *0.05 by *t*-test). (B) Equal amounts of vesicle suspensions were determined by averaging the number of vesicles in 10 electron microscopic images. Each bar presents an average of the number of vesicles in an area of 5.6 × 5.6 *μ*m.

Levels of vesicle production were determined in *P. gingivalis* strains including wild types 33277, its *fimA* mutant (FAE) and *fimR* mutant (FRE) as well as wild-type W83. The W83, FAE, and FRE strains are known as afimbriated strains based on their extremely low expression of long fimbriae (major fimbriae) (Hayashi et al. 2000; Nishikawa and Duncan 2010; Wu and Xie 2010; Zheng et al. 2011). *Porphyromonas gingivalis* FAE carries an insertional mutation in the *fimA* gene encoding a major subunit protein of long fimbriae, while FRE carries a mutation in the *fimR* gene that encodes a response regulator, a member of two component signal transduction system involved in expression of *fim* locus (Hayashi et al. 2000). W83 also lacks fimbrial expression, likely due to a malfunctional FimS, a histidine kinase which acts as a transcriptional activator of *fim* locus (Nishikawa and Duncan 2010). Vesicles were collected from the growth media, and the protein concentration of each vesicle suspension was measured. As shown in Figure1A, although vesicles were detected in the growth media of all four strains, vesicle production in the three afimbriated strains were significantly lower (ranged from 4- to 10-fold) than that found in 33277 growth media, suggesting that the fimbriated strain may produce more vesicles than the afimbriated strains under standard culture conditions. To rule out the possibility that vesicles from W83, FAE, and FRE have a lower protein content, electron microscopy was performed to ensure that vesicle suspension with 0.5 ng of protein concentration derived from each strain contained a similar number of vesicles. The number of vesicles was determined by counting 10 random areas (5.6 × 5.6 *μ*m), and no significant differences in numbers of vesicle suspensions were found among vesicle suspensions (Fig.1B). These data demonstrate a positive correlation between the expression level of FimA and the efficiency of vesicle production in *P. gingivalis*.

**Figure 2:**
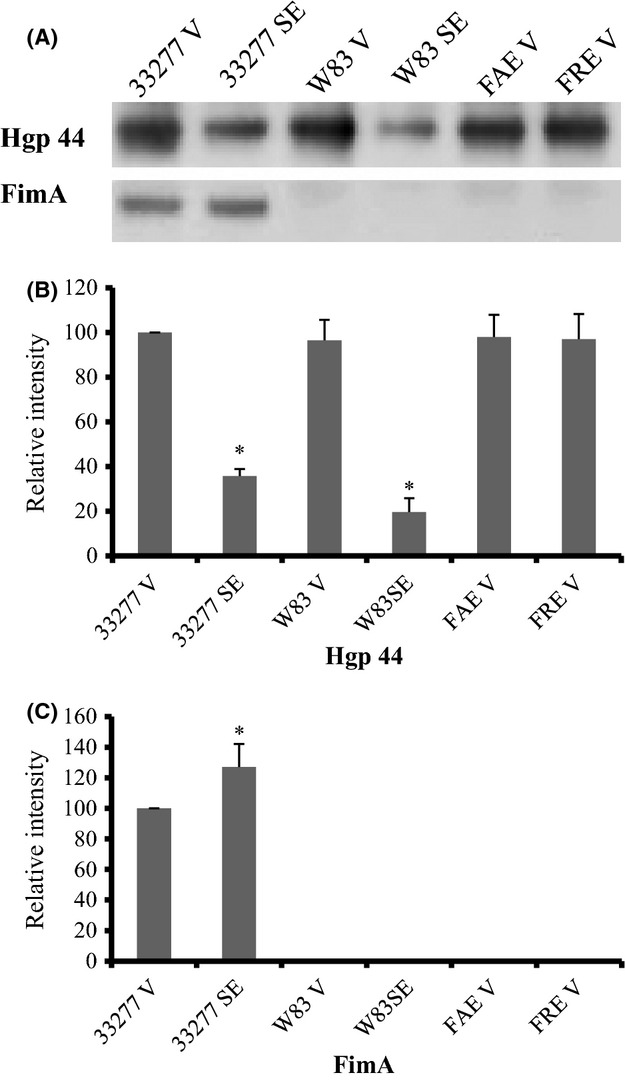
Expression of Hgp44 and FimA in *Porphyromonas gingivalis* vesicles. (A) Levels of FimA and Hgp44 of gingipains in surface extracts (SE) of *P. gingivalis* 33277 or an afimbriated strain (W83), and in vesicles (V) of 33277, W83, *fimA* mutant (FAE), and *fimR* mutant (FRE) were analyzed using western blot analysis with rabbit anti-FimA or rabbit anti-Hgp44 sera. (B, C) Semiquantitation of western blots was conducted with ImageJ software. Each bar represents the relative intensity of (B) Hgp44 and (C) FimA. Values are shown with their standard deviations (*n *=* *3). An asterisk indicates a significant difference between the relative level of Hgp44 or FimA in vesicles or surface extracts of *P. gingivalis* strains compared to those seen in 33277 vesicles (*P <* *0.05 by *t*-test).

We then examined the levels of FimA and gingipains, two well-known virulence factors of *P. gingivalis*, in 0.5 *μ*g of vesicles and compared them with those found in surface extracts (0.5 *μ*g) of the original bacterial cells using western blot analysis with anti-FimA and anti-Hgp44 (a binding domain of gingipains) sera as probes. As expected, FimA protein was only detected in *P. gingivalis* 33277 and in its vesicles, but not in W83 surface extracts or its vesicles, or in vesicles from the *fimA* and *fimR* mutants (FAE and FRE) (Fig.2A). In contrast, a C-terminal adhesive domain of gingipains (Hgp44) was identified in both 33277 and W83 surface extracts as well as in the vesicles of 33277, FAE, FRE, and W83. The protein bands were quantitated using image processing software (ImageJ). As shown in Figure2B, the Hgp44 domain was evidently enriched in the vesicles of all four strains when compared to the level found in surface extracts of 33277 and W83. An approximately threefold increase in Hgp44 levels was observed in 33277 vesicles compared to the level in surface extracts of the originating cells, and a fivefold increase in Hgp44 in W83 vesicles (Fig.2B). In contrast, a 20% reduction was detected in FimA level in 33277 vesicles, compared to the levels in surface extracts of 33277 cells (Fig.2C). These data imply that vesicles production and assembly are regulated in *P. gingivalis*.

### Comparison of protein profiles of vesicles from *P. gingivalis* 33277 and W83

To systematically identify the protein components of *P. gingivalis* vesicles, we tested protein cargo selection in vesicles by comparing the protein compositions of vesicles isolated from *P. gingivalis* 33277 and W83, whose genomes have been sequenced, using LC-MS/MS analyses. A total of 67 proteins were identified in 33277 vesicles (Table S2) with at least two or more unique peptide hits, and 70 identified in W83 vesicles (Table S3). Among the identified proteins, 12 proteins were unique to 33277, and included the minor components of major fimbriae, FimC, D, and E (Table1). Twenty-three proteins were only found in W83, and included a PorT protein and several proteins with a tetratricopeptide repeat (TPR) domain. Consistent with the results of western blot analyses, gingipains were a major component of vesicles from both *P. gingivalis* strains based on the number of peptide spectral counts observed (Table2). FimA (major component of long fimbriae) and Mfa1 (major component of short fimbriae) were abundant in 33277 vesicles, but were sparse in W83 vesicles. Our results revealed differential protein profiles in vesicles from wild-type strains 33277 and W83.

**Table 1:** Unique protein profiles in vesicles derived from *Porphyromonas gingivalis* 33277 versus W83

W83	33277
TPR (tetratricopeptide repeat) domain protein, PG_1385	Uncharacterized protein, PGN_0458
Uncharacterized protein, PG_1795	Uncharacterized protein, PGN_0795
Uncharacterized protein, PG_0026	Immunoreactive 32 kDa antigen, PGN_0290
Prolyl oligopeptidase family protein, PG_1004	Uncharacterized protein, PGN_0291
TPR domain protein, PG_1028	Minor component, FimE
Uncharacterized protein, PG_1786	Minor component, FimD
Uncharacterized protein, PG_2174	Immunoreactive 23 kDa antigen, PGN_0336
Peptidase, M16 family, PG_0196	Minor component, FimC
Peptidyl-prolyl *cis*-*trans* isomerase, PG_2164	Uncharacterized protein, PGN_0288
Uncharacterized protein, PG_1093	Uncharacterized protein, PGN_1823
Uncharacterized protein, PG_0373	Uncharacterized protein, PGN_0654
Uncharacterized protein, PG_1634	FtsK/SpoIIIE family protein, PGN_0487
Uncharacterized protein, PG_0083	
Cobyrinic acid A, CbiA	
Uncharacterized protein, PG_1030	
TonB-dependent receptor, PG_0668	
Phosphoserine aminotransferase, SerC	
Uncharacterized protein, PG_1621	
Uncharacterized protein, PG_0218	
TPR domain protein, PG_1651	
Cysteine peptidase, PG_1788	
PorT protein, PG0751	
TPR domain protein, PG_0449	

**Table 2:** Comparison of the top 20 most abundant proteins identified in *Porphyromonas gingivalis* 33277 and W83 vesicles

Rank^1^	33277	W83
1	Lys-gingipain, kgp	**Arg-gingipain, RgpA**
2	**Arg-gingipain, RgpA**^2^	**Receptor antigen A, RagA**
3	**Por secretion system protein PorV**	**Por secretion system protein PorV**^2^
4	**Arg-gingipain, RgpB**^2^	**Arg-gingipain, RgpB**
5	**Receptor antigen A, RagA**^2^	**Receptor antigen B, RagB**
6	**Peptidylarginine deiminase**	**Peptidylarginine deiminase**
7	**Hemagglutinin protein, HagA**	**Hemagglutinin protein HagA**^2^
8	Major fimbrial subunit protein type-1 FimA^2^	**Immunoreactive 61 kDa antigen**^2^
9	**Receptor antigen B, RagB**^2^	**Uncharacterized protein (PG_1823, PGN_1744)**
10	**Immunoreactive 61 kDa antigen**	**Zinc carboxypeptidase, putative**
11	**Uncharacterized protein (PGN_1744)**	Uncharacterized protein
12	Mfa1 fimbrilin, Mfa1^2^	**Uncharacterized protein (PG_1626, PGN_0477)**
13	**Putative lipoprotein**	**Putative lipoprotein**
14	**Zinc carboxypeptidase, putative**	**35 kDa hemin binding protein**
15	Immunoreactive 23 kDa antigen^2^	**Extracellular protease, putative**
16	**Uncharacterized protein (PGN_0477)**	**Immunoreactive 47 kDa antigen**
17	**Immunoreactive 47 kDa antigen**	Uncharacterized protein
18	**35 kDa hemin binding protein**^2^	Uncharacterized protein
19	Putative uncharacterized protein	Heme-binding protein FetB^2^
20	**Extracellular protease, putative**	Outer membrane protein 41^2^

Bolded proteins were observed in the top 20 (ranked) list for both strains.

1 Rank is based on the number of peptide spectral counts observed for each protein.

2 The protein is abundant in vesicles of this strain.

### Minor components of long fimbriae are associated with invasive activity of *P. gingivalis* vesicles

**Figure 3:**
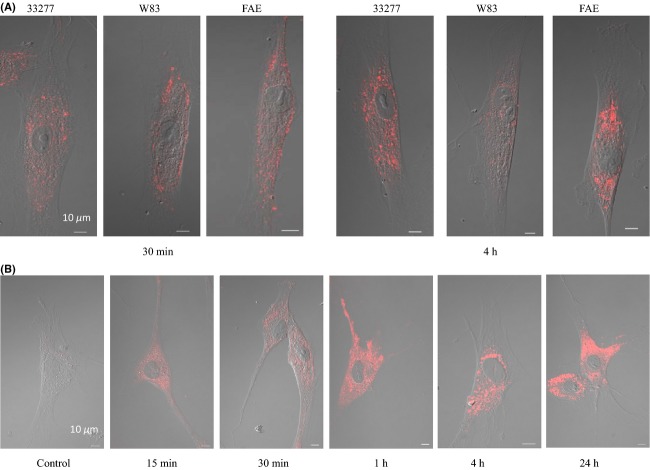
Visualization of intracellular vesicles in human gingival fibroblasts (HGFs) with confocal microscopy. (A) HGFs were grown in a glass bottom dish for 16 h, and then infected with *Porphyromonas gingivalis* vesicles (0.5 *μ*g) isolated from 33277, *fimA* mutant (FAE), and an afimbriated strain (W83) strains. Cells were examined by confocal microscopy at 30 min and again at 4 h. Internalized vesicles were probed with anti-*P. gingivalis* polyclonal antibodies, visualized by Alexa Fluor 546-conjugated anti-rabbit IgG secondary antibody, and presented by differential interference contrast (DIC) imaging. (B) HGFs were treated with or without (control) 33277 vesicles for 15 min to 24 h as shown. As in (A), vesicles were probed with anti-*P. gingivalis* polyclonal antibodies and visualized by Alexa Fluor 546-conjugated anti-rabbit IgG secondary antibody.

Adhesive proteins hemagglutinin A (HagA), FimA, and gingipains (RgpA and Kgp) are reportedly involved in *P. gingivalis* attachment to, and invasion of, host cells (Yilmaz et al. 2002; Chen and Duncan 2004; Zhang et al. 2011; Belanger et al. 2012). We speculated, therefore, that *P. gingivalis* 33277 vesicles, containing all these proteins, would be able to invade HGF with a significantly higher efficiency than W83 or the *fimA* mutant. To test the invasive activity of *P. gingivalis* vesicles, HGFs were exposed to vesicles isolated from 33277, FAE, and W83, and the internalization of vesicles was observed under a confocal microscope. Not surprisingly, vesicles of all three strains were found to invade HGFs, but at different efficiencies (Fig.3A). After exposure to HGFs for 30 min, most of the 33277 vesicles were internalized. We found that while the majority of vesicles from FAE or W83 appeared to be attached to the surfaces of HGFs at 30 min after exposure, only FAE vesicles were internalized by 4 h to the same degree as 33277 vesicles. In contrast at 4 h, far fewer W83 vesicles were observed inside HGFs than 33277 vesicles. These findings indicate different invasive capabilities in terms of rates of internalization, among the three strains. Observation of the internalized vesicles of 33277 for a 24 h duration revealed that the vesicles invaded HGFs by 15 min and then translocated toward the nuclei at the 4 h time point (Fig.3B). These results are in agreement with an earlier report by Furuta et al. (2009b) who demonstrated a sorting process of the internalized vesicles of *P. gingivalis* from early endosomes to lysosomes in HGFs over 24 h. Detachment of HGFs from the poly-l-lysine–coated glass bottom dishes was not observed, and HGFs carrying internalized vesicles remained viable for 24 h after initial exposure under our experimental conditions (Fig.3B).

**Figure 4:**
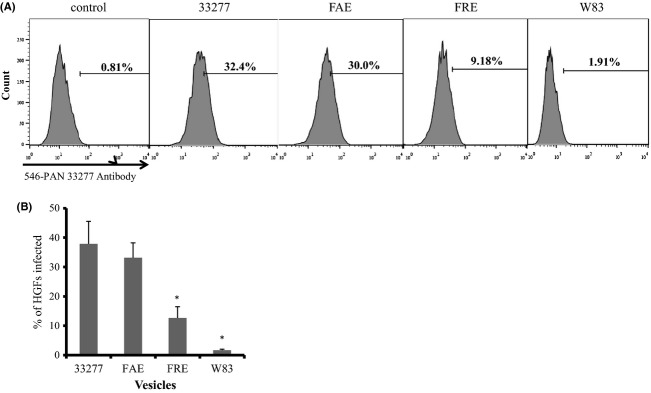
Invasive efficiencies of *Porphyromonas gingivalis* vesicles using human gingival fibroblasts (HGFs). HGFs were infected with *P. gingivalis* vesicles derived from 33277, *fimA* mutant (FAE), *fimR* mutant (FRE), and an afimbriated strain (W83), respectively. Four hours after an initial exposure, the infected HGFs were analyzed by flow cytometry. (A) Representative FACS scatter plots for quantification of the infected HGFs with vesicles of different *P. gingivalis* strains. The percent of infected HGFs is indicated. (B) Bars are shown with standard deviations (*n *=* *3) and represent the percent of HGFs with internalized vesicles. An asterisk indicates a significant difference between the % HGFs infected with 33277 vesicles compared to the % infected using the other *P. gingivalis* strains (*P <* *0.05 by *t*-test).

HGFs with internalized vesicles were quantitated using a flow cytometry assay. Four hours after initial exposure to *P. gingivalis* vesicles, 40% of HGFs were sorted with vesicles from *P. gingivalis* 33277, while only 2% of cells had W83 vesicles (Fig.4A and B). Consistent with this confocal microscopy observation, there was no significant difference in the invasion rates of vesicles isolated from 33277 and its *fimA* mutant, which is not in agreement with a previous study that reported a FimA-dependent entry of human gingival epithelial cells (Furuta et al. 2009b). Thus, we speculated that the minor components of fimbriae, such as FimC, FimD, and/or FimE, are involved in vesicle invasion, since these proteins are known to play a critical role in the adhesive activities of the mature FimA fimbriae in *P. gingivalis* (Nishiyama et al. 2007) and are exclusively found in 33277 vesicles (Table1). Therefore, a *fimR* mutant was tested for its invasive activity. As expected, vesicles from a *fimR* mutant showed a threefold decrease in invasive activity into HGFs compared to those from its parent strain, 33277 (Fig.4A and B).

**Figure 5:**
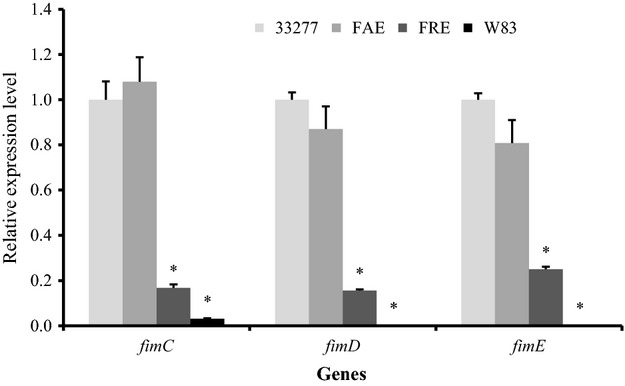
Differential expression of *fimC*, *fimD*, and *fimE* in *Porphyromonas gingivalis* strains. Total RNA was extracted from *P. gingivalis* 33277, *fimA* mutant (FAE), *fimR* mutant (FRE), and an afimbriated strain (W83). The expression of *fimC*, *fimD*, and *fimE* in *P. gingivalis* was measured using a quantitative reverse transcript polymerase chain reaction (qRT-PCR). Each bar represents the relative expression of the gene shown compared to that in 33277 (unit of 1.0) after normalization with the expression level of *glk*. An asterisk indicates a statistically significant difference in expression level of the genes between *P. gingivalis* FAE, FRE, or W83, and 33277 (*t*-test; *P *<* *0.05).

RT-PCR analyses were performed to verify expression levels of *fimC*, *fimD*, and *fimE* in 33277 and its *fimA* and *fimR* mutants. The expression of the *fimC*, *fimD*, and *fimE* genes in the *fimR* mutant was drastically repressed compared to that of its parent strain (Fig.5). In contrast, there was no significant difference in expression levels of these genes in 33277 and the *fimA* mutant, which is consistent with earlier observations showing that the *fimA* gene is monocistronic (Dickinson et al. 1988; Amano et al. 1994). The findings also indicate that there was no polar effect of the mutation in *fimA* on its downstream genes (*fimC*, *fimD*, and *fimE*). Expression of these genes was not detectible in *P. gingivalis* W83. Therefore, a comparison of the expression levels of *fimC*, *fimD*, and *fimE* in *P. gingivalis* strains and their respective vesicle invasive capabilities implicates the minor components of long fimbriae as having a role in vesicle entry into HGFs.

### Invasion and translocation of monolayered HOKs by *P. gingivalis* vesicles

To determine if *P. gingivalis* vesicles can translocate through HOK layers, two-dimensional (monolayer) HOKs cultures in transwell inserts were exposed to vesicles. We found that HOKs stayed stable during the period of 10 days, and assay conditions were noncytotoxic as determined by a Pierce LDH Cytotoxicity Assay Kit (data not shown). Thus permeability of the two-dimensional (monolayer) HOKs cultures was smaller than 1%, after 8 days in culture (data not shown).

**Figure 6:**
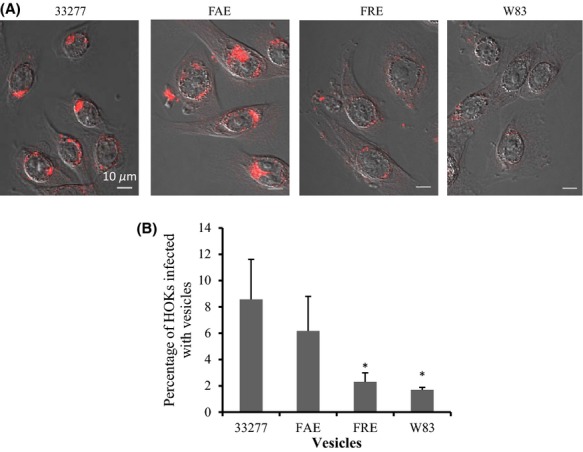
Invasive activity of *Porphyromonas gingivalis* vesicles into human oral keratinocytes (HOKs). (A) Vesicles derived from *P. gingivalis* 33277, *fimA* mutant (FAE), *fimR* mutant (FRE), or an afimbriated strain (W83) were added to transwells covered with a monolayer of HOKs. After a 24 h exposure, HOKs were collected by trypsinization and grown in a glass-bottomed dish for 24 h. *Porphyromonas gingivalis* vesicles were stained with Alex Fluor 546 (red) and observed under a confocal microscope. Infected cells are presented using differential interference contrast (DIC) images. (B) The percent of HOKs infected with vesicles was quantitated using FACS analysis. Data are shown as means ± standard deviations (*n *=* *3). An asterisk indicates a significant difference between the percent of HOKs with internalized vesicles of *P. gingivalis* strains compared to those with 33277 vesicles (*P <* *0.05 by *t*-test).

The uptake of vesicles by HOKs was visualized using a confocal microscopy after a 24 h treatment of vesicles, followed by culturing of the HOKs carrying vesicles for another 24 h in a glass-bottomed dish. In agreement with a previous report (Furuta et al. 2009b), 33277 vesicles were internalized in HOKs and remained in the cytoplasm for 48 h without a significant impairment of the host cells (Fig.6A). Similar to that observed in HGFs, internalization of W83 and FRE vesicles into HOKs was much less than that seen with 33277 and FAE vesicles. Interestingly, vesicles derived from 33277 and FAE appeared to aggregate and form bigger clusters once inside HOKs. This phenomenon likely resulted from the expression of the minor components of fimbriae, since autoaggregation activities were reduced in the *fimC*, *fimD*, and *fimE* mutants which lacked these minor components of the fimbriae, rather than the loss of fimbriae themselves (Nishiyama et al. 2007). FACS was used to quantitate the number of HOKs with internalized vesicles after vesicles translocated through the HOK monolayer. More HOKs were internalized with 33277 and FAE vesicles than with FRE and W83 vesicles (Fig.6B).

**Figure 7:**
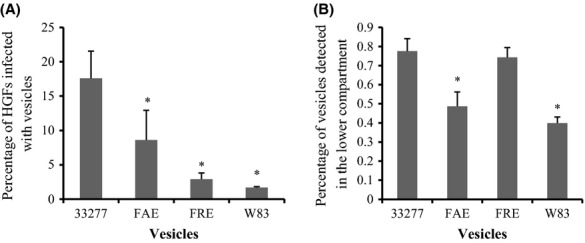
Transepithelial transport of *Porphyromonas gingivalis* vesicles across a monolayer and invasion of vesicles into human gingival fibroblasts (HGFs) in a transwell culture system. (A) The ability of vesicles derived from 33277, *fimA* mutant (FAE), *fimR* mutant (FRE), and an afimbriated strain (W83) to translocate across a monolayer of HOKs was examined using a transwell system and quantitated with enzyme-linked immunosorbent assay (ELISA). Data are shown as means of three independent experiments with standard deviations. An asterisk indicates a significant difference between translocated vesicles of 33277 compared to those with vesicles from other strains (*P <* *0.05 by *t*-test). (B) The percent of HGFs infected by transepithelial vesicles in the wells of a six-well plate is shown. An asterisk indicates a significant difference between the percent of HGFs with internalized vesicles of *P. gingivalis* strains compared to those with 33277 vesicles (*P <* *0.05 by *t*-test).

We then examined translocation of *P. gingivalis* vesicles through two-dimensional HOK cultures. Vesicles were added to the transwells sealed with HOKs, and growth media in the lower compartments were collected by centrifugation 24 h after initial inoculation and quantitated using ELISA. A 40–50% decrease in translocation was found in vesicles derived from W83 and FAE compared to 33277 vesicles (Fig.7A). Translocation rates were similar for 33277 and of FRE vesicles. This was not surprising, as given the lower invasive activity of FRE vesicles into HOKs, there should be more intercellular vesicles in the monolayer of HOKs. W83 vesicles had the lowest translocation activity among the tested vesicles, when their invasive activity into HOKs in the transwell inserts was taken into consideration. When invasive activities of vesicles translocated through the HOK monolayer were examined, we found 50% fewer positive HGFs with internalized FAE vesicles compared to the HGFs with internalized 33277 vesicles (Fig.7B). We postulated, therefore, that this reduction is likely due to the decreased ability of FAE vesicles to translocate through a HOK monolayer.

## Discussion

In this study, we compared the protein profiles of vesicles from two *P. gingivalis* strains, 33277 (fimbriated) and W83 (afimbriated). Most well-known major outer membrane proteins of *P. gingivalis* (Imai et al. 2005) were found to be among the major components of these vesicles. For example, RagA, RagB, Kgp, and Rgp were among the top ranked proteins of 33277 and W83 vesicles. Therefore, we speculated that *P. gingivalis* vesicles function as an extension of the bacterial outer membrane, and are involved in interactions between the organism and its environment. Major subunit proteins (FimA and Mfa1) of long and short fimbriae were abundant components of 33277 but not W83 vesicles, which is consistent with two recent proteomic studies using either 33277 or W83 vesicles alone (Nakao et al. 2014; Veith et al. 2014). It is worthy to note that several TPR such as PG1385 (TprA), PG1028, PG1651, and PG0449 were exclusively found in W83 (Table1). TPR-containing proteins are known to be directly involved in bacterial virulence, such as the translocation of virulence factors into host cells, and adhesion to host cells (Edqvist et al. 2006; Chakraborty et al. 2008). Previous studies, using the mouse subcutaneous infection model, showed that expression of W83 TrpA was upregulated in a mouse subcutaneous chamber, and that the survival rates of mice infected with the *tprA* mutant were significantly higher than those of mice infected with the parent strain W83, suggesting a role of TrpA in *P. gingivalis* virulence (Yoshimura et al. 2008; Kondo et al. 2010) although the mechanism remains to be clarified. Our study also revealed an enrichment of HGP44 (an adhesive domain of gingipains) in vesicles. These enriched or excluded proteins in *P. gingivalis* vesicles support the notion of bacterial vesicles containing selected cargo. Based on a recent report, *P. gingivalis* 33277 vesicles (5–10 *μ*g/mL) caused a detachment of cultured oral epithelial cells, and the detachment activity of the vesicles was gingipain dependent (Nakao et al. 2014). Although the detachment of HGFs or HOKs was not observed with a protein concentration of vesicles (0.5 *μ*g/mL), we found a detachment effect when HGFs were exposed to a higher protein concentration of vesicles (>3 *μ*g/mL) from both 33277 and W83 (data not shown). Therefore, it is conceivable that production of *P. gingivalis* vesicles may be a major mechanism for release and delivery of gingipains into periodontal tissues.

Examination of vesicle production revealed a differential formation and release in *P. gingivalis* strains. A higher level of vesiculation was found in the growth media of 33277 when compared to W83 media. An intriguing finding is that the other two afimbriated strains, the *fimA* mutant and the *fimR* mutant, also produced significantly fewer vesicles than its parent strain 33277. A recent study by Kerr et al. (2014) using transmission electron microscopy, showed a copious number of outer membrane vesicles on the surfaces of many *P. gingivalis* strains including 33277, while a few vesicles were observed on the surface of W83. Although these authors and our laboratory examined vesicle production at different sites (in the growth media vs. on the surface of bacterial cells), the parallel results suggest that FimA production or its regulatory system is likely involved in vesicle biogenesis in *P. gingivalis*. Interestingly, requirement of the synthesis of flagella proteins for vesicle production was recently found in *E. coli* W3110 (Manabe et al. 2013). In fact, the deletion or overexpression of several membrane-associated proteins were known to affect vesiculation in many gram-negative bacteria, which likely results from an alteration of envelope structure and/or decreased membrane stability (Baker et al. 2014).

One of important features of *P. gingivalis* is its ability to invade and survive within host cells (Tribble and Lamont 2010). In this study, we demonstrated that *P. gingivalis* vesicles invaded both HGFs and HOKs. However, the invasive activities varied between vesicles derived from 33277 and W83, which is consistent with the invasive activities observed in their originating cells (Wayakanon et al. 2013). Since FimA was far more abundant in 33277 vesicles than in W83 vesicles, we speculated that a decreased invasive activity would be found in vesicles from the *fimA* mutant, as we showed previously that the *fimA* mutant lost 90% of its invasive activity into HGFs (Chaudhuri et al. 2014). Surprisingly, vesicles from this mutant showed a similar invasive activity as 33277, while vesicles from the *fimR* mutant showed a significantly decreased invasive activity, due to reduced expression of the *fim* locus that encodes minor components (FimC, FimD, and FimE) of long fimbriae. These findings are consistent with a previous report that fimbriae purified from the *fimC*, *fimD*, and *fimE* mutants showed reduced binding activities to *Streptococcus oralis* and to fibronectin and type I collagen, suggesting that these minor components of fimbriae play critical roles in their adhesive activities (Nishiyama et al. 2007). We note that minor components of bacterial fimbriae are generally considered to act as adhesins or are responsible for fimbrial assembly. In the case of vesicles, the minor components are more likely to be adhesins, since mature fimbrial structures were not observed on the surfaces of vesicles isolated from all *P. gingivalis* strains tested.

Bacterial vesicles, based on their small size and their adhesive and proteolytic activities, are known to spread more readily in tissues than in bacterial cells (Kuehn and Kesty 2005). *Porphyromonas gingivalis* vesicles were shown in this study to penetrate through a monolayer of HOKs and then invade HGFs. According to the current state of knowledge, *P. gingivalis* cells spread in periodontal tissues via intercellular translocation established by actin-dependent membrane protrusions or through intercellular junctions, which require gingipain activity (Andrian et al. 2004; Yilmaz et al. 2006). Takeuchi et al. (2011) also showed an epithelial translocation of *P. gingivalis* cells that was mediated by an endocytotic recycling pathway . Although *P. gingivalis* vesicles were able to enter human epithelial cells by an endocytic pathway and were sorted to lysosomal compartments (Furuta et al. 2009b), there was no evidence of the vesicles trafficking out the cells through recycling endosomes. We observed that considerable amount of *P. gingivalis* vesicles remained within HOKs 48 h after the initial invasion, and that vesicles of the *fimR* mutant (with a limited invasive activity) were also able to penetrate the monolayer of HOKs at a similar level as 33277 vesicles. Therefore, spreading of *P. gingivalis* vesicles is in part through an intercellular transport mechanism.

In summary, the observations reported here provide evidence of a differential production, protein assembly, and invasive activity of vesicles derived from fimbriated and afimbriated strains of *P. gingivalis*. This work suggests that there is an opportunity to reduce *P. gingivalis* virulence and vesiculation via regulating its fimbrial expression and to also identify distinctive vesicles which may be modulated and utilized as a much safer vaccine than their parent bacterial cells.

## Supporting Information

**Table S1.** Primers used in this study. **Table S2.** Proteins identified in *Porphyromonas gingivalis* 33277 vesicles by mass spectrometer. **Table S3.** Proteins identified in *Porphyromonas gingivalis* W83 vesicles by mass spectrometer.
