# Supplementary material for: Fimbriae-mediated outer membrane vesicle production and invasion of Porphyromonas gingivalis
Source: Microbiologyopen. 2014 Dec 18;4(1):53–65. doi: 10.1002/mbo3.221 (PMC4335976; doi:10.1002/mbo3.221)
Supplement: Supplementary file 1 — Table S1. Primers used in this study. Table S2. Proteins identified in Porphyromonas gingivalis 33277 vesicles by mass spectrometer. Table S3. Proteins identified in Porphyromonas gingivalis W83 vesicles by mass spectrometer. [file mbo30004-0053-sd1.docx]

**TABLE S1** Primers used in this study.

| Primer name | Primer sequence | Application |
| --- | --- | --- |
| fimC187F | cctttcacgatcagtgctca | qPCR for *fimC* gene |
| fimC187R | taagagtcgccgtagccatt |  |
| fimD162F | gtataccggtggatggttcg | qPCR for *fimD* gene |
| fimD162R | gcgtctgatcgtctgaatga |  |
| fimE196F | gatctctgtggccgatgatt | qPCR for *fimE* gene |
| fimE196R | tccctttgttccaggaattg |  |
| PG16S-F | tgtagatgactgatggtgaaa | qPCR for *16S rRNA* gene |
| PG16S-R | actgttagcaactaccgatgt |  |

| **Table S2. Proteins identified in *P. gingivalis* 33277 vesicles by mass spectrometer.** | | | |
| --- | --- | --- | --- |
| Protein description | Filtered Spectra^a^ | *P*-value | FDR |
| Lys-gingipain, Kgp | 243 | 0.0664345 | 0.0509603 |
| Arg-gingipain, RgpA | 189 | 0.0646619 | 0.0399056 |
| Receptor antigen A, RagA | 142 | 1.42E-11 | 4.03E-05 |
| Arg-gingipain, RgpB | 96 | 0.135047 | 0.0799408 |
| Major fimbrial subunit protein type-1, FimA | 93 | 6.13E-13 | 4.03E-05 |
| Probable peptidylarginine deiminase, PGN_0898 | 80 | 0.055783 | 0.0348946 |
| Receptor antigen B, RagB | 70 | 3.47E-12 | 4.03E-05 |
| Por secretion system protein, PorV | 60 | 3.39E-16 | 4.03E-05 |
| Mfa1fimbrilin, Mfa1 | 57 | 1.30E-09 | 4.03E-05 |
| Hemagglutinin protein, HagA | 50 | 0.00125433 | 0.0014851 |
| Putative uncharacterized protein, PGN_0154 | 40 | 0.0419918 | 0.0322964 |
| Putative uncharacterized protein, PGN_0335 | 36 | 0.0108634 | 0.00849953 |
| Immunoreactive 61 kDa antigen, PGN_0152 | 33 | 0.00385955 | 0.0027281 |
| Probable immunoreactive 23 kDa antigen, PGN_0482 | 30 | 0.013669 | 0.00865043 |
| Putative uncharacterized protein, PGN_1744 | 29 | 0.00245598 | 0.00189794 |
| 35 kDa hemin binding protein, PGN_0659 | 26 | 0.0150794 | 0.00988726 |
| Putative uncharacterized protein, PGN_0693 | 23 | 0.0161456 | 0.00990843 |
| Putative uncharacterized protein, PGN_0458 | 21 | 0.000105343 | 0.000103836 |
| Putative uncharacterized protein, PGN_0289 | 21 | 0.00118056 | 0.00114559 |
| Immunoreactive 47 kDa antigen, PGN_0852 | 20 | 1 | 0.449208 |
| Putative uncharacterized protein, PGN_0477 | 20 | 0.594514 | 0.276081 |
| Immunoreactive 46 kDa antigen, PGN_1767 | 18 | 0.00309202 | 0.0027281 |
| NAD-specific glutamate dehydrogenase, Gdh | 16 | 0.261494 | 0.144822 |
| Putative uncharacterized protein, PGN_0795 | 15 | 0.00179086 | 0.00150057 |
| Putative uncharacterized protein, PGN_1557 | 15 | 0.82477 | 0.367647 |
| Trypsin like proteinase, PrtT | 13 | 0.193233 | 0.111237 |
| Putative uncharacterized protein, PGN_2080 | 13 | 0.812337 | 0.367263 |
| Putative uncharacterized protein, PGN_0156 | 13 | 0.0227247 | 0.0174684 |
| Putative uncharacterized protein, PGN_1611 | 12 | 0.0984455 | 0.0539511 |
| Probable lysyl endopeptidase, PGN_1416 | 12 | 0.0296585 | 0.0174684 |
| Immunoreactive 32 kDa antigen, PGN_0290 | 11 | 0.00939792 | 0.00696283 |
| Putative uncharacterized protein, PGN_0123 | 11 | 0.279286 | 0.144822 |
| C-terminal domain of Arg-and Lys-gingipain proteinase, PGN_0295 | 9 | 0.0309787 | 0.0192647 |
| Putative uncharacterized protein, PGN_1816 | 8 | 0.167745 | 0.0992747 |
| Thiol protease, PGN_0900 | 8 | 0.341999 | 0.170446 |
| Putative uncharacterized protein, PGN_0129 | 8 | 1 | 0.449208 |
| Putative uncharacterized protein, PGN_0558 | 8 | 0.430818 | 0.211018 |
| Outer membrane protein 41, PGN_0729 | 8 | 0.0128605 | 0.00865043 |
| Putative uncharacterized protein, PGN_0291 | 7 | 0.0521875 | 0.0322964 |
| Minor component, FimE | 7 | 0.0521875 | 0.0322964 |
| Minor component, FimD | 6 | 0.0917321 | 0.0539511 |
| 28 kDa outer membrane protein Omp28, PGN_0122 | 6 | 1 | 0.449208 |
| Ferritin, PGN_0604 | 6 | 0.375315 | 0.211018 |
| Outer membrane protein 40, PGN_0728 | 6 | 0.00681665 | 0.00599519 |
| Immunoreactive 23 kDa antigen, PGN_0336 | 5 | 0.165317 | 0.0972476 |
| Minor component, FimC | 5 | 0.165317 | 0.0972476 |
| Immunoreactive 84 kDa antigen, PGN_0509 | 5 | 1 | 0.449208 |
| DNA protection during starvation protein, Dps | 5 | 1 | 0.449208 |
| Por secretion system protein, PorQ | 5 | 0.512563 | 0.237744 |
| Heme-binding protein, FetB | 5 | 4.09E-05 | 0.00010754 |
| Putative uncharacterized protein, PGN_0288 | 4 | 0.303471 | 0.170446 |
| Putative uncharacterized protein, PGN_0296 | 4 | 0.658074 | 0.367263 |
| Putative uncharacterized protein, PGN_0322 | 4 | 0.475433 | 0.243669 |
| TonB-dependent receptor, PGN_0741 | 4 | 0.0199674 | 0.0126692 |
| Putative uncharacterized protein, PGN_1735 | 3 | 0.152055 | 0.0898025 |
| Glyceraldehyde 3-phosphate dehydrogenase type I, PGN_0173 | 3 | 0.152055 | 0.0898025 |
| Putative uncharacterized protein, PGN_1808 | 3 | 0.00014868 | 0.000149347 |
| Putative uncharacterized protein, PGN_1823 | 2 | 0.535243 | 0.255783 |
| Putative uncharacterized protein, PGN_0654 | 2 | 0.535243 | 0.255783 |
| FtsK/SpoIIIE family cell division protein, PGN_0487 | 2 | 0.535243 | 0.255783 |
| TonB-linked receptor, Tlr, PGN_0683 | 2 | 1 | 0.449208 |
| Putative uncharacterized protein, PGN_0471 | 2 | 1 | 0.449208 |
| Putative uncharacterized protein, PGN_1010 | 2 | 0.627433 | 0.28333 |
| Putative uncharacterized protein, PGN_0860 | 2 | 0.363328 | 0.188331 |
| Putative uncharacterized protein, PGN_1129 | 2 | 0.200586 | 0.111237 |
| Putative uncharacterized protein, PGN_1514 | 2 | 0.000542899 | 0.0011659 |
| Putative heat shock-related protease, HtrA | 2 | 9.58E-05 | 0.00010754 |

^a^ The number of peptide spectral counts observed from each protein.

| **Table S3. Proteins identified in *P. gingivalis* W83 vesicles by mass spectrometer.** | | | |
| --- | --- | --- | --- |
| Protein description | Filtered Spectra ^a^ | *P*-value | FDR |
| Lipoprotein, RagA | 229 | 5.29E-46 | 6.75E-06 |
| Hemagglutinin protein, HagE | 139 | 1.19E-10 | 6.75E-06 |
| Lipoprotein, RagB | 118 | 3.37E-19 | 6.75E-06 |
| Uncharacterized protein, PG_0027 | 118 | 0.0353872 | 0.0387297 |
| Gingipain, RgpB | 71 | 0.000169082 | 0.000164063 |
| Hemagglutinin A, HagA | 59 | 0.834754 | 0.593591 |
| Immunoreactive 61 kDa antigen, PG_2102 | 41 | 0.712261 | 0.471102 |
| Uncharacterized protein, PG_1823 | 36 | 1 | 0.593591 |
| Peptidylarginine deiminas,e PG_1424 | 32 | 3.36E-11 | 6.75E-06 |
| Uncharacterized protein, PG_2106 | 28 | 0.334956 | 0.298254 |
| Uncharacterized protein, PG_1881 | 24 | 0.000395554 | 0.00039363 |
| Putative extracellular protease, PG_0553 | 19 | 0.443156 | 0.357269 |
| Lipoprotein, putative, PG_2105 | 19 | 1 | 0.593591 |
| Heme-binding protein, FetB | 18 | 0.0469795 | 0.0459452 |
| Uncharacterized protein, PG_1626 | 17 | 0.0462832 | 0.0459452 |
| Outer membrane protein 40, PG_0694 | 16 | 0.178579 | 0.177213 |
| Outer membrane protein 41, PG_0695 | 15 | 0.51599 | 0.286795 |
| Uncharacterized protein, PG_0448 | 13 | 0.0361065 | 0.0380019 |
| HtrA protein | 12 | 0.0568003 | 0.0522034 |
| Immunoreactive 47 kDa antigen, PG_1374 | 12 | 0.113608 | 0.0893827 |
| Uncharacterized protein, PG_1889 | 11 | 0.00432577 | 0.00543457 |
| TPR domain protein, PG_1385 | 10 | 0.00766459 | 0.0277586 |
| Uncharacterized protein, PG_1795 | 10 | 0.00766459 | 0.0277586 |
| PDZ domain protein, PG_1726 | 10 | 0.058852 | 0.0619133 |
| Uncharacterized protein, PG_0409 | 10 | 0.809898 | 0.471102 |
| Outer membrane lipoprotein, Omp28 | 9 | 0.382528 | 0.319963 |
| TonB-dependent receptor, putative, PG_0707 | 9 | 1 | 0.593591 |
| Uncharacterized protein, PG_0026 | 8 | 0.0251473 | 0.0277586 |
| Uncharacterized protein, PG_2216 | 8 | 0.155963 | 0.17243 |
| Zinc carboxypeptidase, putative, PG_0232 | 8 | 3.00E-08 | 6.75E-06 |
| Prolyl oligopeptidase family protein, PG_1004 | 6 | 0.0871864 | 0.0737607 |
| Lipoprotein, putative, PG_1835 | 6 | 1 | 0.593591 |
| Uncharacterized protein, PG_0602 | 6 | 1 | 0.593591 |
| HmuY protein | 6 | 0.558557 | 0.35837 |
| TPR domain protein, PG_1028 | 5 | 0.164191 | 0.177213 |
| Lipoprotein, putative, PG_0188 | 5 | 0.411953 | 0.357269 |
| Uncharacterized protein, PG_1382 | 5 | 0.709448 | 0.430546 |
| Glyceraldehyde 3-phosphate dehydrogenase, type I GapA | 5 | 1 | 0.593591 |
| Ferritin, Ftn | 5 | 0.532492 | 0.35837 |
| NAD-specific glutamate dehydrogenase, Gdh | 5 | 0.00369606 | 0.00441262 |
| Thioredoxin, putative, PG_0616 | 5 | 5.75E-06 | 6.75E-06 |
| Uncharacterized protein, PG_1786 | 4 | 0.155424 | 0.117682 |
| Uncharacterized protein, PG_2174 | 4 | 0.155424 | 0.117682 |
| Peptidase, M16 family, PG_0196 | 4 | 0.155424 | 0.117682 |
| Peptidyl-prolyl cis-trans isomerase, PG_2164 | 4 | 0.155424 | 0.117682 |
| Uncharacterized protein, PG_0491 | 4 | 0.653994 | 0.406627 |
| Uncharacterized protein, PG_1341 | 4 | 1 | 0.593591 |
| Uncharacterized protein, PG_2168 | 4 | 0.0756409 | 0.0737607 |
| Uncharacterized protein, PG_0654 | 4 | 9.65E-06 | 6.75E-06 |
| Uncharacterized protein, PG_0937 | 3 | 1 | 0.593591 |
| Uncharacterized protein, PG_0217 | 3 | 0.688102 | 0.430546 |
| DNA protection during starvation protein, Dps | 3 | 0.447123 | 0.37737 |
| Immunoreactive 84 kDa antigen, PG_1604 | 3 | 0.447123 | 0.37737 |
| Uncharacterized protein, PG_2172 | 3 | 0.032142 | 0.0387297 |
| Uncharacterized protein, PG_1093 | 2 | 0.520153 | 0.338532 |
| Uncharacterized protein, PG_0373 | 2 | 0.520153 | 0.338532 |
| Uncharacterized protein, PG_1634 | 2 | 0.520153 | 0.338532 |
| Uncharacterized protein, PG_0083 | 2 | 0.520153 | 0.338532 |
| Cobyrinic acid A,C-diamide synthase, CbiA | 2 | 0.520153 | 0.338532 |
| Uncharacterized protein, PG_1030 | 2 | 0.520153 | 0.338532 |
| TonB-dependent receptor, PG_0668 | 2 | 0.520153 | 0.338532 |
| Phosphoserine aminotransferase, SerC | 2 | 0.520153 | 0.338532 |
| Uncharacterized protein, PG_1621 | 2 | 0.520153 | 0.338532 |
| Uncharacterized protein, PG_0218 | 2 | 0.520153 | 0.338532 |
| TPR domain protein, PG_1651 | 2 | 0.520153 | 0.338532 |
| Putative cysteine peptidase, PG_1788 | 2 | 0.520153 | 0.338532 |
| PorT protein | 2 | 0.520153 | 0.338532 |
| TPR domain protein, PG_0449 | 2 | 0.520153 | 0.338532 |
| Thiol protease/hemagglutinin PrtT, PG_1427 | 2 | 0.224445 | 0.298254 |
| Internalin-related protein, PG_0350 | 2 | 0.00124627 | 0.00116486 |

^a^ The number of peptide spectral counts observed from each protein.
